# Polyvinylidene Fluoride (PVDF) and Nanoclay Composites’ Mixed-Matrix Membranes: Exploring Structure, Properties, and Performance Relationships

**DOI:** 10.3390/polym17081120

**Published:** 2025-04-20

**Authors:** Rund Abu-Zurayk, Nour Alnairat, Haneen Waleed, Mohammed Q. Al-Khaial, Aya Khalaf, Ayat Bozeya, Duaa Abu-Dalo, Sojoud Al-Yousef, Razan Afaneh

**Affiliations:** 1 Hamdi Mango Center for Scientific Research, The University of Jordan, Amman 11942, Jordan; 2 Nanotechnology Center, The University of Jordan, Amman 11942, Jordan; alnairatnour@gmail.com; 3 Chemical Engineering Department, Jordan University of Science and Technology, Irbid 22110, Jordan; hwmohammad21@eng.just.edu.jo; 4 College of Pharmacy, University of Al Maarif, Al-Ramadi 31001, Al Anbar, Iraq; mohammed.qahtan@uoa.edu.iq; 5 Allied Sciences Department, Faculty of Arts and Sciences, Al-Ahliyya Amman University, Amman 19328, Jordan; a.khaled@ammanu.edu.jo; 6 Institute of Nanotechnology, Jordan University of Science and Technology, Irbid 22110, Jordan; aabouzieh@just.edu.jo; 7 Faculty of Pharmacy, Basic Pharmaceutical Science Department, Middle East University, Amman 11831, Jordan; d.abudalo@meu.edu.jo; 8 Chemical Engineering Department, School of Engineering, The University of Jordan, Amman 11942, Jordan; sojoudalyousef@gmail.com; 9 Department of Nanoscience, Joint School of Nanoscience and Nanoengineering, University of North Carolina at Greensboro, Greensboro, NC 27401, USA; raafaneh@uncg.edu

**Keywords:** Ag-Nanoclay, ZnO-Nanoclay, roughness, tensile strength, antifouling properties, wastewater treatment

## Abstract

Polyvinylidene fluoride (PVDF) membranes have become a favored choice for membrane filtration because of their outstanding mechanical characteristics, chemical resistance, thermal stability, and ease of handling. Nevertheless, the hydrophobic nature of PVDF membranes can result in fouling, which diminishes their efficiency over time. This study explores the impact of ZnO-Nanoclay on the properties and performance of mixed matrix membranes made from polyvinylidene fluoride (PVDF) at different loading percentages (0, 1, and 3 wt%). The ZnO-Nanoclay nanoparticles were synthesized using environmentally friendly methods, characterized, and blended into PVDF matrices via a solution-casting technique, resulting in a series of membranes. The synthesized nanoparticles were analyzed using Scanning Electron Microscopy (SEM) and X-ray diffraction (XRD). The resulting mixed-matrix membranes underwent comprehensive analyses to assess their structure and surface properties, employing SEM, XRD, Atomic Force Microscopy (AFM), and contact-angle measurements. Furthermore, tensile, antibacterial, and barrier properties were evaluated. Integrating ZnO-Nanoclay into PVDF membranes greatly improves antifouling properties, achieving inhibition rates of 99.92% at a clay-loading percentage of 3 wt% and increasing water-flux rates by 16% compared to pure PVDF membranes at 1 wt%. In addition, ZnO-Nanoclay nanoparticles significantly boost the mechanical properties of PVDF membranes, enhancing maximum strength by 500% at 3 wt% loading. This study examines the interplay between the structure, properties, and performance of mixed-matrix membranes by comparing different PVDF membranes that were mixed with different nanoclay composites, providing significant insights into improving these membranes through the incorporation of nanoclay composites to enhance their overall properties and effectiveness.

## 1. Introduction

Water scarcity is a growing concern worldwide, with billions of people facing the challenge of inadequate access to clean and safe water. Rapid population growth, urbanization, and industrialization exacerbate this critical issue, placing immense pressure on existing water sources and treatment infrastructure [1]. In response to this urgent challenge, there is a pressing need for innovative water-treatment technologies that can effectively address water scarcity and ensure the availability of safe water for all [2,3].

Water treatment encompasses diverse technologies to purify water from various sources, including wastewater, seawater, and contaminated groundwater [4,5]. Removing contaminants and impurities from water is essential to making it safe for consumption and other applications. Among the diverse range of water-treatment methods available, adsorption is widely recognized for its effectiveness in removing pollutants from water [6]. In addition, membrane filtration is a key water-treatment technology that utilizes semipermeable membranes to separate water from contaminants [7]. Membrane filtration processes include microfiltration, ultrafiltration, nanofiltration, and reverse osmosis, each characterized by the size of the pores in the membrane and the types of contaminants they can remove. Due to its efficiency and versatility, membrane filtration is widely used in desalination, wastewater treatment, and reusable water production. By selectively allowing water molecules to pass through while retaining contaminants, membrane filtration can effectively remove particles, bacteria, viruses, and other impurities from water, producing clean and safe water [8].

Polyvinylidene fluoride (PVDF) membranes have emerged as a preferred material for membrane filtration due to their excellent mechanical properties, chemical resistance, thermal stability, and ease of processing [9]. PVDF membranes are commonly used in ultrafiltration and microfiltration applications, where they effectively separate particles, bacteria, and macromolecules from water. However, the hydrophobic nature of PVDF membranes can lead to fouling, reducing their effectiveness over time. Researchers have turned to nanotechnology to address this issue and enhance the performance of PVDF membranes [10].

Nanoparticles such as titanium dioxide (TiO_2_) [11], zinc oxide (ZnO) [12,13,14], Zirconium oxide (ZrO_2_) [15] graphene oxide (GO) [16,17,18], silver (Ag) [19,20] and nanoclay [21] have been found to enhance different properties such as the permeability, selectivity, mechanical strength, and antifouling properties of polymeric membranes made of PVDF.

Utilizing the combined advantages of PVDF’s natural characteristics alongside the specialized functions of nanoparticles, these membranes present a practical approach for treating diverse water sources [22]. The performance improvements offered by distinct types of nanoparticles vary, influenced by their unique effects on structure and properties. Among the numerous nanofillers examined, zinc oxide (ZnO) is particularly noteworthy for its exceptional antibacterial properties and hydrophilicity, significantly improving membrane antifouling and water-purification capabilities. However, directly adding ZnO to polymer matrices presents challenges like nanoparticle agglomeration and poor dispersion, impacting membrane uniformity and longevity. To address these issues, this study utilizes ZnO-Nanoclay as a hybrid nanofiller. Nanoclay is an effective supporting matrix, promoting the uniform distribution of ZnO nanoparticles, boosting mechanical strength, and enhancing interfacial compatibility with the PVDF matrix. This synergistic pairing mitigates the hydrophobicity and fouling issues associated with PVDF membranes, enhancing their water-treatment performance. To our knowledge, this is the first study to explore the integration of ZnO-Nanoclay as a multifunctional nanofiller within PVDF membranes, highlighting its significant potential.

This work also offers novel insights into how fillers influence performance by systematically examining the effects of green-synthesized Ag-Nanoclay and ZnO-Nanoclay composites on membrane structure, properties, and efficiency. The data for PVDF–Ag-Nanoclay membranes were previously published by our group [23] and are included here for comparative purposes. The present work provides a comprehensive analysis, discussions, and deep insights into the structure–performance relationships.

This study presents a novel approach to PVDF membrane design by integrating green-synthesized nanoclay hybrid fillers with metal/metal oxide nanoparticles. It establishes a clear structure–performance relationship, showing how the distribution, interaction, and morphology of Ag-Nanoclay and ZnO-Nanoclay fillers affect membrane properties, providing a new framework for creating high-performance membranes for water treatment.

## 2. Materials and Methods

### 2.1. Materials

To prepare ZnO-Nanoclay, banana peels, zinc acetate dihydrate from Sigma Aldrich, USA (density 1.735 g/cm^3^, Sigma-Aldrich, St. Louis, MO, USA), and sodium hydroxide were utilized. The nanoparticle composites were created using a modified nanoclay (montmorillonite) (Sigma–Aldrich, Merck, Darmstadt, Germany) along with deionized water (DW).

To prepare the PVDF membrane, we utilized polyvinylidene fluoride (PVDF) (Kyner 460), N, N-dimethylacetamide (DMAA) (Tedia, Fairfield, OH, USA), and polyvinylpyrrolidone (PVP) (Sigma-Aldrich, Beijing, China).

For the antibacterial test, we used agar, nutrient agar, peptone meat extract, soybean peptone, casein peptone (Liofilchem, Roseto degli Abruzzi, Italy); nutrient broth (Oxoid, Mumbai, India); D-glucose (JHD, Shantou, China), lecithin (Riedel deHaen AG Seelze–Hannover, Germany); sodium chloride (Lobachemie, Mumbai, India) and Tween 80 (Polysorbate-80) (ICI, Londin, UK), buffer saline (W/O Calcium, W/O Magnesium) (Euroclone, Milan, Italy), and *Escherichia coli* (*E. coli*) (ATCC no. 8739).

### 2.2. Methods

#### 2.2.1. Membrane Preparation

Ag-Nanoclay was previously synthesized following the detailed methodology described in our previous research [23], using *Paronychia argentea Lam* (PA) extract as a reducing and stabilizing agent, nanoclay, and silver nitrate (AgNO_3_) as a precursor. The PVDF-Ag-Nanoclay membranes were previously cast using the phase-inversion technique, with 0 wt%, 1 wt%, and 3 wt% Ag-Nanoclay loadings, following the detailed methodology described in Abu-Zurayk et al. (2024) [23].

ZnO-Nanoclay was prepared as follows: 10 g of banana peel was stirred in 100 mL deionized water at 80 °C for one hour. The solution was then filtered three times using muslin cloth and Whatman No. 1 filter paper, and stored at 4 °C for later use [24]. Solution A involved adding 1 mL of banana-peel extract to 20 mL of distilled water. Solution B consisted of dissolving 1.8 g of zinc acetate in 150 mL of distilled water, then diluting to 180 mL with distilled water. Solution A was gradually mixed into Solution B while stirring at 1000 RPM and maintaining 60 °C for 20 min. Afterward, 0.25 g of nanoclay was added and stirred for 30 min. The pH was raised to 12 using a 2M sodium hydroxide solution. The mixture was stirred for 3 h at 80 °C; then, the supernatant was filtered from the precipitate, and the product was calcined at 400 °C for 2 h in a muffle furnace.

**Scheme 1:**
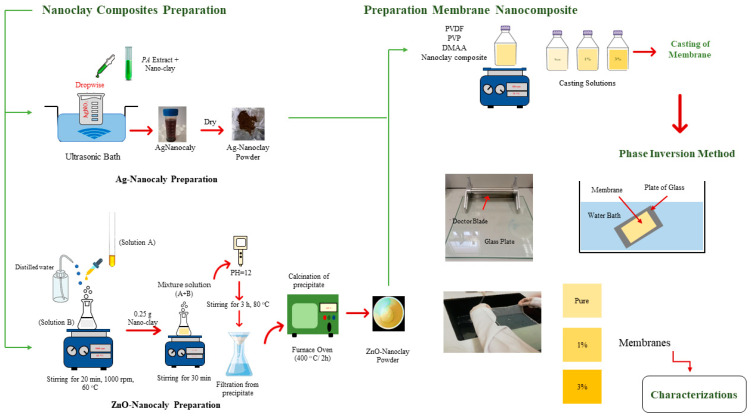
Methodology for preparation of Ag-Nanoclay composite (previously prepared in [23]), ZnO-Nanoclay composite, and PVDF nanocomposite membranes with different nanoclay composite loadings (0 wt%, 1 wt%, 3 wt%).

PVDF-ZnO-Nanoclay membranes were cast using the phase-inversion technique, combining ZnO-Nanoclay (0.11 or 0.33 g) with 38.17 g of dimethylacetamide (DMAA), 11 g of PVDF, and 0.5 g of polyvinylpyrrolidone (PVP) while stirring at 100 rpm for 6 h at 70–80 °C. The mixture was then degassed and cast into films with 1 wt% and 3 wt% ZnO-Nanoclay loading. Pure PVDF membranes were made similarly, without ZnO-Nanoclay. The preparation procedure is summarized in Scheme 1.

#### 2.2.2. Characterization of Nanoclay Composites

Nanoparticle size and shape were analyzed with a Quanta SEM FEG 450, Netherlands. A Rigaku Ultima IV instrument from Japan was used for XRD analysis. The assessment employed a Cu K X-ray tube at 20 mA and 40 kV, 3000°/min speed, and 0.02 as the size of the step. This procedure spanned a range from 3° to 90° to evaluate the structure of nanoclay composites.

#### 2.2.3. Characterization of PVDF Membranes

##### Structure and Surface Properties

SEM from Quanta FEG 450, Netherlands, was used to examine the membranes’ surface morphology. The Gravimetric method was used to calculate the porosity. Samples were collected from each membrane type and were soaked in deionized water until saturation. Then, filter paper was used to remove water from the samples’ surfaces, and the initial weights of the samples were recorded (*m*_1_). The weights of the membranes were subsequently documented (*m*_2_) following a drying period of 48 h at ambient temperature. Equation (1) was employed to compute the porosity of the membranes. (*ε*)%:

(1) $$\varepsilon =\frac{\left(m_{1}-m_{2}\right)/\rho H_{2}O}{\frac{m_{1}-m_{2}}{\rho H_{2}O}+m_{2}/\rho P}$$

where *ε*: porosity, *m*_1_: sample weight (wet); *m*_2_: sample weight (dry); *ρH*_2_*O*: water density (1.0 g/cm^3^); and *ρP*: polymer density (1.74 g/cm^3^ for PVDF).

Contact angle was used to evaluate the hydrophilicity of the membranes’ surfaces using Biolin Scientific’s Attension with the sessile drop technique.

The membranes’ surface topologies were examined using Atomic Force Microscopy (AFM) (AIST-NT SmartSPM 1000), which functioned in non-contact mode using NSC14/Al BC. The SI tips were operated at 160 kHz resonant frequency, with a scanning rate that varied between 0.3 kHz and 1 kHz.

##### Mechanical Properties

The tensile properties of the membrane were evaluated utilizing a BMT-E Series (BESMAK, Ankara, Turkey) testing machine. A tensile speed of 5 mm/min was employed.

##### Antibacterial Properties

According to our previous paper [23], the antibacterial efficiency of nanoclay composites has been tested against *E. coli* using the liquid medium method. Bacterial suspensions (~6 × 10^6^ cells/mL) were treated with nanoclay composites in nutritional broth. Negative controls used pure broth, while positive controls had bacteria without nanoclay. After overnight incubation at 37 °C, bacterial growth was measured at 600 nm using an ELISA reader. The inhibitory efficiency (%) was estimated as follows:

(2) $$\mathrm{Inhibition}\;\mathrm{Efficiency}=\frac{Control\;OD-Tested\;OD}{Control\;OD}\times \mathrm{100}\%$$

The *control OD* was from bacteria without nanoclay composites, whereas the measured OD was from bacteria with nanoclay composites [25].

According to the international standard procedure (ISO 22196 [26]), the antibacterial properties of membranes were tested:

For preparation of culture media, 28 g of nutrition agar and 13 g of nutritional broth were each dissolved in 1 L of DW. For the SCDLP broth, combine soybean peptone (3 g), casein peptone (17 g), disodium hydrogen phosphate (2.5 g), sodium chloride (5 g), glucose (2.5 g), lecithin (1 g), and non-ionic surfactant (7 g) in 1 L of DW. Three samples of PVDF nanocomposite membranes (2.2 × 2.2 cm^2^) and six control samples of pure PVDF membranes were prepared. Half of the PVDF-Pure samples were washed with 10 mL of the SCDLP buffer, diluted in saline buffer to 10-fold, and 100 μL from each dilution was plated on agar and incubated at 37 °C for 24 h. The surviving bacteria from the SCDLP-washed samples underwent a similar dilution, plating, and analysis process.

Equation (3) was used to calculate the viable bacteria number per cm^2^ (N) for each tested sample:

*N* = (100 × *C* × *D* × *V*)/*A* (3)

where

(*N*): viable bacteria number (cm^2^); (*C*): average plate count; (*D*): dilution factor; (*V*): volume of SCDLP buffer (mL); and (*A*): surface area (mm^2^).

The sterilization ratio (R) was determined as [27]:

(4) $$R=\frac{\left(A-B\right)}{A}\times 100\%$$

where

(*A*): viable bacteria per cm^2^ on untreated PVDF-Pure membranes; and (*B*): viable bacteria per cm^2^ on PVDF-nanocomposite membranes.

##### Barrier Properties

The dead-end cell (HP4750; Sterlitech, Auburn, WA, USA) measured the pure water flux across membranes with an area of 14.6 cm^2^ at a pressure of 1.0 bar.

Permeability flux (*j_w_*) was calculated using Equation (5):

(5) $$j_{w}=\frac{v}{A\cdot \Delta T}$$

where *v* (L) is the volume of water permeate; *A* (m^2^) is the effective area of the membrane; and Δ*T* (h) is the permeability time.

## 3. Results

### 3.1. Nanoclay Composite Characterization

#### 3.1.1. SEM

**Figure 1:**
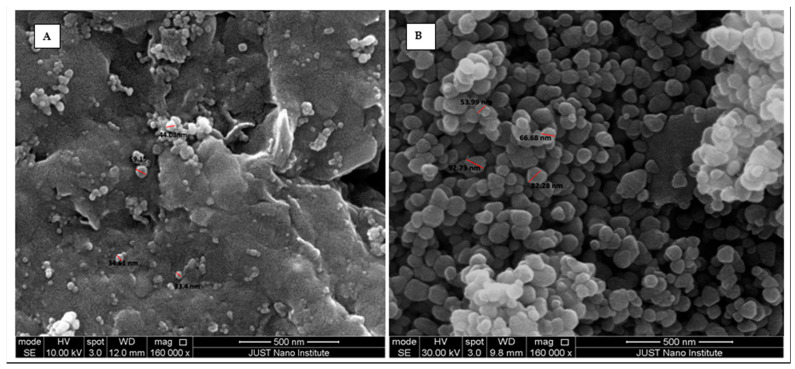
SEM images of Ag-Nanoclay (**A**) and ZnO-Nanoclay (**B**) at a magnification of ×160,000; scale bar = 500 nm. Bright regions indicate the presence of metal nanoparticles.

The morphology, dimensions, and distribution of metallic nanoparticles on the surfaces of the nanoclay layers within the synthesized Ag-Nanoclay and ZnO-Nanoclay composites were evaluated using SEM. Figure 1A illustrates the nanoclay layers, which are partially covered by a low concentration of silver nanoparticles (AgNPs) that are unevenly distributed across their surfaces, exhibiting sizes that range from 20 to 60 nm, which was reported previously [23]. On the other hand, Figure 1B demonstrates a high particle concentration of well-ordered ZnO nanoparticles with larger size ranges of 40–120 nm, almost entirely covering the surfaces of the nanoclay layers. This difference in size is expected, since the nanoparticles were prepared using two different techniques; the Ag-Nanoclay nanoparticle was prepared using chemical reduction, while ZnO-Nanoclay was prepared using chemical precipitation. It is known that nanoparticles synthesized by the chemical reduction method can be strictly controlled mainly due to surfactants, which stabilize the nanoparticle’s size [28,29], which explains the smaller size of AgNPs than ZnO nanoparticles. ZnO had a higher particle concentration on the nanoclay layers than AgNP concentration on the nanoclay layers. This may be related to the higher compatibility between ZnO and the nanoclay surface due to the presence of oxygen, and consequently, an OH group, which forms hydrogen bonds or interacts electrostatically with the functional groups on the nanoclay surface [30]—unlike the AgNPs surface, which has lower interaction with the nanoclay layers. Ye et al. (2015) synthesized ZnO nanosheet/montmorillonite photocatalysts using an alkaline hydrolysis synthesis method in situ without calcining [31]. Similarly to our study, the ZnO nanosheets were densely packed on the nanoclay support; however, their diameters ranged between 200 and 900 nm [31].

#### 3.1.2. XRD

**Figure 2:**
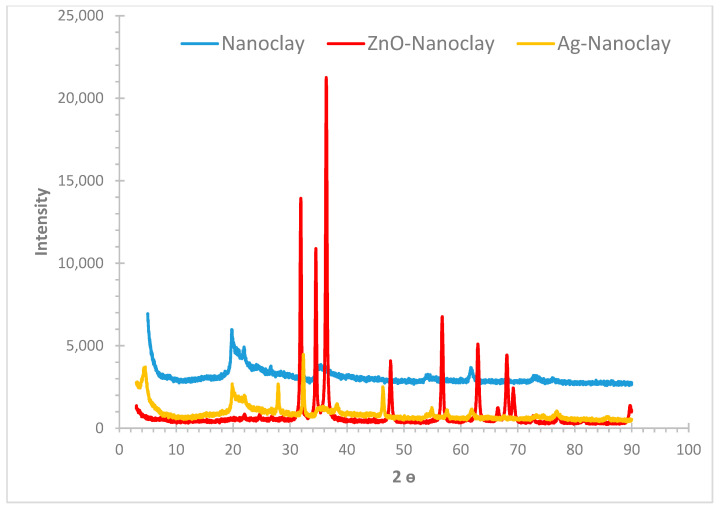
XRD patterns of nanoclay, Ag-Nanoclay, and ZnO-Nanoclay nanoparticles.

Figure 2 demonstrates the XRD peaks for the nanoclay and synthesized nanoclay composites of Ag-Nanoclay and ZnO-Nanoclay.

As previously reported [23], in Ag-Nanoclay, the peaks at 2θ = 4.5°, 20.0°, 26.5°, 36.3°, and 54.7° correspond to the planes (111), (110), (210), (124), and (144) of nanoclay. Meanwhile, the diffraction peaks with 2θ values of 38.1°, 44.1°, 27.8°, and 78.1° are linked to Bragg’s reflections (111), (200), (220), and (311), indicating that Ag nanoparticles exhibit a face-centered cubic structure. Additionally, the peaks at 32.3° and 46.3° may be associated with the organic phases present in the plant [22,32,33,34].

Conversely, for the ZnO nanoparticles, the XRD peaks recorded at 2θ = 31°, 34.6°, 36.3°, 47.7°, 56.6°, 63.1°, 66.5°, 68.1°, and 69.2° correspond to the (1 0 0), (0 0 2), (1 0 1), (1 0 2), (1 1 0), (1 0 3), (2 0 0), (1 1 2), and (2 0 1) reflections, respectively [35]. The presence of diffraction peaks (100), (002), and (101) at 2θ values of 31°, 34.6°, and 36.3°, respectively, confirms the formation of the hexagonal wurtzite structure of ZnO [36]. Ahmad et al. (2022) also reported the formation of a hexagonal wurtzite structure of ZnO nanoparticles prepared using the sol–gel technique [37].

The higher intensities of peaks of ZnO-Nanoclay compared to those of Ag-Nanoclay can be related to different crystallinity and crystal morphology [38] due to the calcination step during ZnO-Nanoclay synthesis, since the higher temperatures during calcination provide enough thermal energy for the porous or poorly crystalline state to transition to a well-ordered crystalline structure. In addition, this can be an indication of larger crystal size, as found by [36]’s study during the synthesis of ZnO nanoparticles, in which the crystallite size was increased at calcination temperatures higher than 300 °C, resulting in the strengthening of the ZnO phase.

### 3.2. Membrane Characterization

#### 3.2.1. Structure and Surface Properties

##### SEM

**Figure 3:**
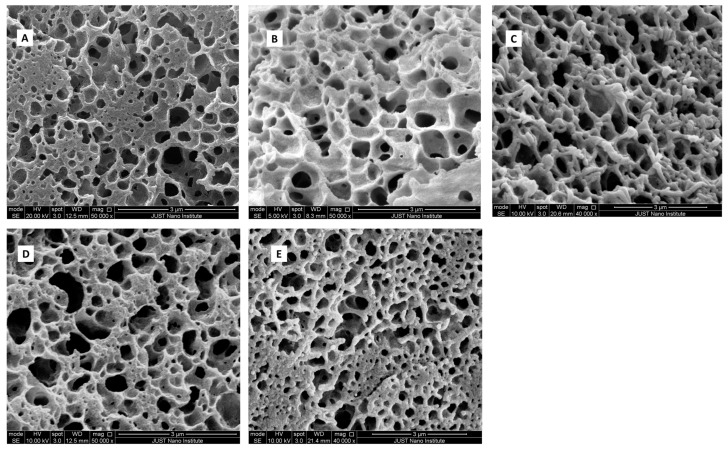
Cross-sectional SEM images of PVDF membranes: (**A**). Pure PVDF; (**B**). PVDF-1%Ag-Nanoclay; (**C**). PVDF-1%ZnO-Nanoclay; (**D**). PVDF-3%Ag-Nanoclay; (**E**). PVDF-3%ZnO-Nanoclay. Scale bar = 3 µm.

Figure 3 presents a cross-section of PVDF membranes (see Figure 3A–E) exhibiting pore sizes and shapes, with pore size being determined using Jmicrovision and shown in Table 1. They did not undergo significant changes through the addition of 1 wt% of Ag-Nanoclay (Figure 3B) and 3 wt% of Ag-Nanoclay (Figure 3D), as reported before [23]. However, the introduction of ZnO-Nanoclay resulted in a pore-size increase with the addition of 1 wt% (Figure 3C) and a decrease with the addition of 3 wt% (Figure 3E). This observation can be attributed to the relatively larger size and higher concentration of ZnO nanoparticles within the nanoclay layers, as opposed to the smaller Ag nanoparticles located within the nanoclay layers. These two variables exert opposing influences: at low loading (1 wt%), the larger-sized nanoparticles significantly contribute to the formation of larger voids within the polymer matrix by disrupting the polymer chains to a greater extent during the membrane casting process; conversely, at higher loading (3 wt%), the increased concentration of nanoparticles limit the mobility of the polymer chains [39], causing them to pack more closely together and reducing the pore sizes between them. Similar results were found by the authors of [40], who found that using larger nanoparticle sizes caused an increase in the pore size of membranes. Another point to mention here is that ZnO-Nanoclay, due to its surface chemistry (presence of hydroxyl groups), interacts more strongly with PVDF than Ag-Nanoclay, further influencing pore structure.

**Figure 4:**
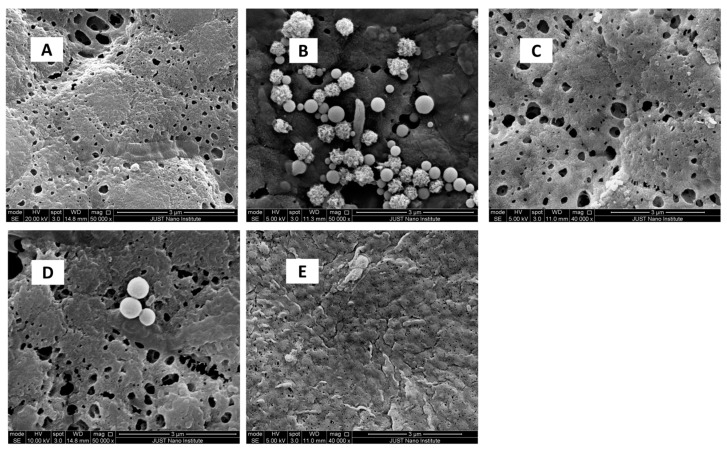
Surface SEM images of PVDF membranes: (**A**). Pure PVDF; (**B**). PVDF-1%Ag-Nanoclay; (**C**). PVDF-1%ZnO-Nanoclay; (**D**). PVDF-3%Ag-Nanoclay; (**E**). PVDF-3%ZnO-Nanoclay. Scale bar = 3 µm.

Figure 4A–E presents SEM images of the membrane surfaces, revealing similar pore-size trends as observed in cross-sectional analysis (Figure 3). The addition of ZnO-Nanoclay has a pronounced effect on pore morphology, where lower loadings (1 wt%) result in increased pore size due to polymer-chain disruption, while higher loadings (3 wt%) lead to reduced pore size due to restricted polymer mobility and the densification of the matrix. Additionally, nanoparticle distribution differs between Ag-Nanoclay and ZnO-Nanoclay. As shown in Figure 4B,D, Ag-Nanoclay nanoparticles are visible on the membrane surface, which was reported in a previous publication [23]. In contrast, ZnO-Nanoclay particles (Figure 4C,E) are not observed externally, suggesting they are embedded within the polymer matrix. This distribution pattern can be attributed to the stronger affinity of ZnO nanoparticles for the PVDF matrix, likely due to their surface hydroxyl (-OH) groups, which promote hydrogen bonding and electrostatic interactions with the polymer chains. In contrast, the lower compatibility between Ag-Nanoclay and PVDF results in weaker interactions, causing Ag nanoparticles to migrate toward the membrane surface during phase inversion. This finding further supports the idea that nanoparticle–polymer compatibility is crucial in determining their spatial distribution and overall membrane morphology. Roshani et al. reached a similar conclusion by creating PVDF membranes incorporating PS/ZnO nanocomposites. Their SEM images demonstrate that the nanoparticles established strong interactions with the PVDF polymer chains, embedding themselves within the membrane’s inner structure [41].

##### Porosity

Table 2 shows the porosity of the prepared membranes, indicating that the incorporation of nanoparticles results in a moderate increase in porosity. This effect is due to the inclusion of hydrophilic nanoparticles in the casting solution, which enhances the exchange rate between solvents and non-solvents during phase inversion. The presence of these nanoparticles facilitates water diffusion into the polymeric film, promoting pore formation and overall porosity improvement [42,43]. However, a decrease in porosity was noted at higher ZnO-Nanoclay concentrations (3 wt%) compared to 1 wt%. This reduction is mainly due to increased solution viscosity as more ZnO nanoparticles are added. Higher viscosity slows the solvent–non-solvent exchange rate, decreasing phase-separation efficiency and lowering porosity at elevated nanomaterial concentrations. This trend is consistent with previous studies on nanocomposite membranes [44,45,46,47]. Interestingly, the viscosity-related reduction in porosity was observed only in PVDF-ZnO-Nanoclay membranes and not in PVDF-Ag-Nanoclay membranes. This difference can be attributed to the higher concentration of ZnO nanoparticles within the nanoclay layers and their stronger interactions with the PVDF matrix. Due to their surface chemistry and functional groups, ZnO nanoparticles exhibit enhanced hydrogen bonding and electrostatic interactions with PVDF, resulting in increased viscosity. In contrast, Ag-Nanoclay demonstrates weaker interactions with PVDF, leading to improved nanoparticle dispersion and minimal impact on viscosity. This explanation is supported by findings from [48], further confirming the influence of nanoparticle–polymer interactions on membrane morphology and porosity.

Ardeshiri et al. (2018) investigated the impact of ZnO nanoparticles on the porosity of PVDF membranes [49]. Their findings indicated that at low concentrations of ZnO nanoparticles, the porosity of PVDF membranes increased, but it decreased at higher concentrations. In line with our findings, they attributed this behavior to the increase in viscosity associated with higher nanoparticle concentrations [49].

##### Contact Angle and Surface Roughness

PVDF membranes were tested for hydrophilicity and roughness. Table 3 shows data on contact angles and average roughness.

The pure PVDF a membrane exhibited contact angle of 53°, indicating a hydrophilic surface. With the addition of nanoparticles, the contact angle increased in all cases, suggesting a shift toward greater hydrophobicity, although the membranes remained hydrophilic (i.e., contact angles stayed below 90°). This increase can primarily be attributed to changes in surface roughness, as supported by previous studies [50,51]. However, the effect of nanoparticle incorporation on surface roughness varied between PVDF-Ag-Nanoclay and PVDF-ZnO-Nanoclay membranes. As reported in [23], in PVDF-Ag-Nanoclay membranes, roughness increased with the addition of Ag-Nanoclay (Table 2), mainly due to the accumulation of Ag nanoparticles on the membrane surface, as confirmed by SEM images (Figure 4B,D). The increased surface roughness resulted in a higher contact angle, as surface protrusions inhibit the spreading of water droplets. Conversely, PVDF-ZnO-Nanoclay membranes displayed different behavior. The increased contact angle in these membranes is attributed not to higher roughness but rather to the well-dispersed ZnO-Nanoclay nanoparticles, which created a more uniform membrane surface. Furthermore, ZnO nanoparticles served as nucleation sites, improving the crystallinity of PVDF by promoting polymer-chain alignment and tighter molecular packing during membrane formation, in which similar results were found by Li et al. (2019) [52]. Using ZnO nanoparticles without nanoclay produced varying results. Hong et al. (2012) investigated the impact of ZnO nanoparticles on the contact angle and roughness of PVDF [12]. They discovered that the increasing ZnO nanoparticles led to a decrease in the contact angle, while the surface roughness decreased with a low loading of ZnO nanoparticles; however, it increased at higher loadings due to the anticipated accumulation of the nanoparticles [12].

Thus, this increased crystallinity reduced surface roughness, as shown in Table 2, and contributed to the observed rise in contact angle. These findings emphasize the dual influence of nanoparticles on membrane wettability: Ag-Nanoclay increases roughness-driven hydrophobicity, whereas ZnO-Nanoclay enhances crystallinity-driven hydrophobicity.

#### 3.2.2. Mechanical Properties

**Figure 5:**
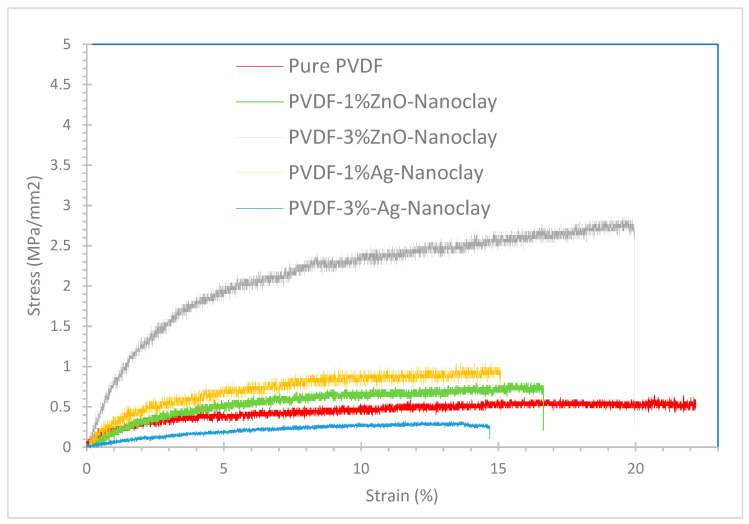
Tensile stress–strain curves of pure PVDF and PVDF nanocomposite membranes—the influence of nanoclay composite type and loading on maximum strength and elongation at break.

Figure 5 illustrates the tensile properties of the PVDF membranes, emphasizing the different effects of Ag-Nanoclay and ZnO-Nanoclay at varying loadings. The inclusion of Ag-Nanoclay enhanced the tensile strength of the PVDF membranes at 1 wt%. Still, at 3 wt%, the strength decreased due to nanoparticle agglomeration, which disrupts the polymer matrix and creates stress-concentration points, leading to premature failure as reported before in [23]. In contrast, ZnO-Nanoclay improved the mechanical properties of the PVDF membranes at both 1 wt% and 3 wt%, with particularly significant enhancement at 3 wt%, where the maximum tensile strength increased from 0.5 MPa/mm^2^ to 3 MPa/mm^2^, representing a 500% improvement. Hwang et al. (2011) reported similar findings, noting that using only nanoclay improved mechanical properties significantly, with maximum tensile strength enhancement reaching 400% [53]. This substantial enhancement confirms the more homogeneous dispersion of ZnO-Nanoclay within the PVDF matrix, which prevents defect formation and promotes better stress transfer. Regardless of the type of nanoparticle, the elongation at break decreased with the addition of nanoparticles. This reduction is attributed to the intrinsic brittleness of nanoclay, which restricts polymer-chain mobility and reduces the material’s ability to deform under stress [23]. ZnO nanoparticles without nanoclay were found to increase the tensile strength and elongation at break of PVDF at 0.1 wt% loading, as found by He et al. (2011) [54].

#### 3.2.3. Antibacterial Properties

**Figure 6:**
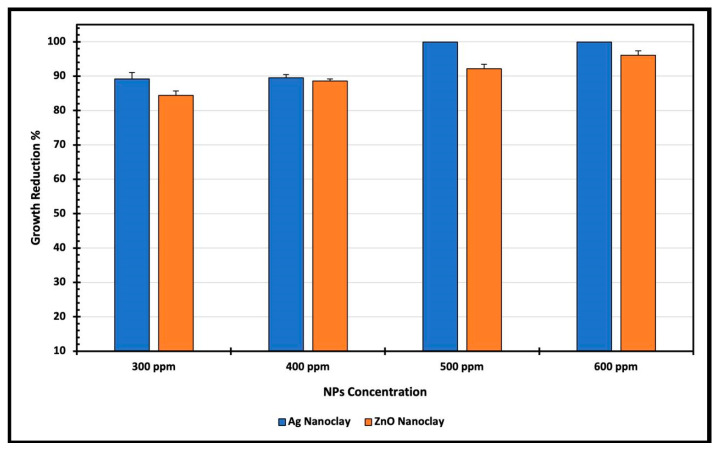
Inhibition rates of Ag-Nanoclay and ZnO-Nanoclay nanoparticles against *E. coli* bacteria at different concentrations.

The results in Figure 6 show that both nanoparticles have strong antibacterial effectiveness against *E. coli*, with slight differences in effectiveness. The optimal Ag-Nanoclay concentrations were 500 and 600 ppm, achieving inhibition rates of 99.9%. In contrast, ZnO-Nanoclay was most effective at 600 ppm, with an inhibition rate of 96.1%.

AgNPs attached to the nanoclay exhibited a spherical shape and a high surface area-to-volume ratio, which improves their capacity to bond with the cell walls of the bacteria, enhancing their antibacterial effectiveness [55]. AgNPs can modify the phosphor-tyrosine profiles of bacterial peptides, affecting the signal-transduction pathways that restrict microbial growth and proliferation. Their antibacterial activity is dose dependent and independent of the bacteria’s antibiotic resistance mechanisms. When *E. coli* cells are treated with AgNPs, the nanoparticles aggregate in the bacterial membranes, increasing membrane permeability and ultimately leading to cell death [56,57,58].

Other research in the literature observed comparable effects of Ag-Nanoclay composites on E. coli. The clay serves as a physically stable surface for the nucleation of AgNPs. Concurrently, its parallel-stacked layered structure facilitates the diffusion-controlled antibacterial action of the Ag that precipitates in situ [59]. Such composites are effective, cost-efficient, and environmentally friendly antibacterial agents [60].

In general, the antibacterial activity of ZnO-Nanoclay nanocomposites involves a complex process, including producing ROS such as –OH, –O_2_−, ^1^O₂, and H₂O₂ [61,62,63,64,65,66,67]. ZnO NPs induce bacterial damage and cell death by releasing H₂O₂ and ROS, which interact with DNA, lipids, and proteins [63].

The literature on ZnO-Nanoclay composites as effective antibacterial materials found similar results. Such composites were prepared using sol–gel techniques at pH levels 7 and 11 and calcination temperatures of 250 and 400 °C [57] or by a simple hydrothermal method using non-ionic surfactants, specifically Tween 20 [68].

Table 4 describes the antibacterial efficiency of the PVDF membranes against *E. coli*. The results indicate that PVDF membranes with Ag-Nanoclay exhibit superior antibacterial performance at lower concentrations than PVDF membranes with ZnO-Nanoclay. However, both nanocomposite membranes demonstrate excellent antibacterial activity at higher concentrations.

This can be explained based on the SEM results, which showed that some Ag-Nanoclay nanoparticles are available on the PVDF membrane’s surface. In contrast, ZnO-Nanoclay nanoparticles are mainly embedded within the PVDF matrix. The availability of nanoparticles on the surface provides an advantage for antibacterial efficiency since they are in direct contact with bacterial cells. Any release of antibacterial agents such as Ag^+^, Zn^+2^, or ROS will be directly into the surrounding environment.

The antibacterial efficiency of Ag and ZnO nanocomposites has been demonstrated in the literature. Wang and coworkers formulated a palygorskite-Ag nanocomposite incorporated within a thin film nanocomposite membrane with remarkable antibacterial properties. AgNPs incorporated in polydopamine-coated palygorskite dramatically improved the antibacterial activity of the membrane against *E. coli* [69]. In another study, Gabriel and coworkers prepared AgNPs on montmorillonite/chitosan films through a photochemical approach. Antibacterial tests showed that all AgNP-based nanocomposite films effectively inhibited the growth of E. coli, showing a significant probability of antibacterial utilization [70,71,72]. Another study showed that electrospun filter membranes incorporating halloysite nanotubes and ZnO NPs into polycaprolactone nanofibers had very good results for antibacterial activity, achieving 95.9% against *E. coli* [73].

#### 3.2.4. Barrier Properties

Table 5 presents the water flux of PVDF nanocomposite membranes, which is influenced by multiple factors, including pore size, porosity, surface roughness, and hydrophilicity. Due to the interplay of these parameters, water flux does not follow a consistent trend across different nanoparticle types and loadings.

The PVDF-3%Ag-Nanoclay membrane exhibited the highest water flux, which can be attributed to higher surface roughness and increased porosity. These factors facilitate water passage by reducing hydraulic resistance. Conversely, the PVDF-3%ZnO-Nanoclay membrane significantly reduced water flux, primarily due to the combined effects of lower pore size, reduced surface roughness, and decreased hydrophilicity. The smaller pores and smoother surface limit water transport, while reduced hydrophilicity further decreases water affinity, leading to lower permeability. For the other PVDF membranes, the water flux showed moderate fluctuations depending on the balance between pore structure, surface morphology, and wettability. The opposing effects of these parameters resulted in either a slight increase or decrease in permeability.

The literature presented varied findings. Ayyaru et al. (2020) reported a 48% increase in water flux for the GO-ZnO/PVDF membrane compared to pure PVDF, attributed to its greater hydrophilicity [74]. Conversely, He et al. (2011) discovered that adding ZnO nanoparticles to the PVDF membrane decreased water flux due to reduced porosity [54].

## 4. Structure–Properties–Performance Relationship

Table 6 summarizes how structural characteristics (pore size, roughness, hydrophilicity, nanoparticle distribution) influence the properties (mechanical strength, porosity, hydrophilicity) and subsequently affect the performance (antibacterial activity, water flux) of the PVDF nanocomposite membranes.

Compared to previously reported studies, the individual incorporation of silver (Ag) or zinc oxide (ZnO) nanoparticles into polyvinylidene fluoride (PVDF) membranes has demonstrated significant improvements in both properties and performance. For instance, Ag nanoparticles have been shown to enhance the hydrophilicity and antifouling performance of the PVDF membranes [75]. In another study, Ag nanoparticles exhibited increased water flux and effective antibacterial properties [76]. Similarly, integrating ZnO nanoparticles has been found to enhance the hydrophilicity of the membranes, increase the average pore size, improve water permeability, and provide resistance to bacterial adhesion and biofouling [12,77,78]. Additionally, nanoclay alone has been shown to maintain high membrane porosity (approximately 80%) while significantly increasing mechanical strength, demonstrating its effectiveness as a reinforcement material [53].

Building on these individual advantages, the present study introduces a novel approach that synergistically combines nanoclay with Ag or ZnO nanoparticles in PVDF membranes. This hybrid nanofiller strategy offers multifunctional benefits, allowing for the simultaneous enhancement of antibacterial activity, mechanical robustness, surface morphology, and permeability. The green-synthesized nanoclay composite significantly increased membrane porosity and water flux while exhibiting strong antibacterial performance, making it suitable for applications requiring high throughput and resistance to biofouling. Conversely, ZnO-nanoclay composites produced smoother membrane surfaces and significantly improved mechanical strength, making them ideal for pressure-driven filtration processes. This work highlights the synergistic effects of hybrid nanofillers. It establishes a clear relationship between structure and performance, providing a sustainable and rational design pathway for engineering high-performance membranes tailored to diverse water-treatment needs.

## 5. Conclusions

This study presents a green synthesis of two types of nanoparticles, Ag-Nanoclay and ZnO-Nanoclay, which were added to PVDF membranes by solution casting. Two loading percentages of nanoparticles were used: 1 wt% and 3 wt%. Studying different systems of PVDF membranes shows that the structure, properties, and performance of PVDF membranes can be affected by adding nanoparticles. Ag-Nanoclay nanoparticles were found on the surface of PVDF membranes. Their addition mainly increased the porosity and decreased the hydrophilicity for both loadings, with a higher effect at 3 wt% loading. The surface roughness of PVDF membranes was also affected. Ag-Nanoclay enhanced the antibacterial properties regardless of their wt% contents, while the water flux was significantly enhanced at 3 wt% content due to enhancement in porosity and surface roughness. Based on that, Ag-Nanoclay nanoparticles are recommended for those applications in which high water flux is required or high biofouling is expected.

On the other hand, ZnO-Nanoclay nanoparticles were not seen on the surface of PVDF membranes, which caused a smoother surface. In addition, they caused lower pore size and lower hydrophilicity, resulting in lower antibacterial efficiency and lower water flux through the PVDF membrane. However, the more distributed and well-ordered ZnO nanoparticles on the nanoclay layers significantly enhanced the mechanical properties. Based on that, ZnO-Nanoclay nanoparticles are recommended for applications requiring high pressure during filtration.

Overall, this work illustrates how the synergistic combination of functional nanoparticles with nanoclay supports can strategically tailor PVDF membrane properties, offering an environmentally friendly and versatile method for designing application-specific membranes to address advanced water-treatment challenges.

## Figures and Tables

**Table 1:** Pore size in PVDF membranes using cross-sectional images.

Sample	Average Pore Diameter (nm)
Pure PVDF	295.2
PVDF-1%Ag-Nanoclay	314.5
PVDF-3%Ag-Nanoclay	310.2
PVDF-1%ZnO-Nanoclay	373.5
PVDF-3%ZnO-Nanoclay	242.3

**Table 2:** Average porosity (%) of PVDF membranes.

Sample	Average Porosity (%)
PVDF	77.8 ± 1.7
PVDF-1%Ag-Nanoclay	79.4 ± 2.3
PVDF-3%Ag-Nanoclay	82.4 ± 0.4
PVDF-1%ZnO-Nanoclay	80.9 ± 1.3
PVDF-3%ZnO-Nanoclay	79.6 ± 2.5

**Table 3:** Contact angle and average roughness of PVDF membranes.

Sample	Contact Angle (°)	Average Roughness (nm)
PVDF	53⁰	14.4
PVDF-1%Ag-Nanoclay	55⁰	29.3
PVDF-3%Ag-Nanoclay	66⁰	24.2
PVDF-1%ZnO-Nanoclay	63⁰	13.4
PVDF-3%ZnO-Nanoclay	79⁰	11.7

**Table 4:** PVDF membrane sterilization ratio.

Sample	Sterilization Against *E. coli*
PVDF-1%Ag-Nanoclay	99.98%
PVDF-3%Ag-Nanoclay	99.98%
PVDF-1%ZnO-Nanoclay	61.54%
PVDF-3%ZnO-Nanoclay	99.92%

**Table 5:** Pure water flux of PVDF membranes.

Sample	Water Flux Compared to Pure PVDF Membrane (%)
PVDF-1%Ag-Nanoclay	−7.8
PVDF-3%Ag-Nanoclay	+48.2
PVDF-1%ZnO-Nanoclay	+16.1
PVDF-3%ZnO-Nanoclay	−12.0

**Table 6:** Structure–properties–performance interactions.

Parameter	Nanocomposite	Structure	Properties	Performance
Pore Size	PVDF-Ag-Nanoclay	Small pores (310.2–314.5 nm), surface roughness (29.3 nm, 24.2 nm roughness)	Increased hydrophobicity (Contact angle increased by 13^0^ compared to pure PVDF)	Enhanced antibacterial activity due to higher nanoparticle exposure on surface (99.98% sterilization)
	PVDF-ZnO-Nanoclay	Larger pores at 1 wt% (373.5 nm), smaller pores at 3 wt% (242.3 nm)	Increased crystallinity, lower surface roughness at 3wt% (11.7 nm roughness)	Reduced water flux at 3 wt% (−12%), antibacterial activity due to ROS generation (99.92% sterilization)
Porosity	PVDF-Ag-Nanoclay	Higher porosity at 3 wt% (82.4%), increased voids	Higher porosity facilitates water diffusion	Higher water flux (+48%), but reduced mechanical strength at higher loading (−44%)
	PVDF-ZnO-Nanoclay	Lower porosity at 3 wt%, (79.6) reduced voids	Lower porosity limits water flow	Reduced water flux at 3 wt% (−12%), enhanced mechanical strength at 3 wt% (+500%)
Nanoparticle Distribution	PVDF-Ag-Nanoclay	Nanoparticles are mostly on the surface	Higher nanoparticle loading leads to agglomeration at 3 wt%	Enhanced mechanical strength at 1 wt% (+108%), reduced at 3 wt% (−44%) due to agglomeration
	PVDF-ZnO-Nanoclay	Well-dispersed nanoparticles within the polymer matrix	Better interaction with PVDF, increased viscosity at higher loading	Enhanced mechanical strength at both loadings (+77%, +500%), better distribution of nanoparticles
Surface Roughness	PVDF-Ag-Nanoclay	Increased roughness (29.3 nm, 24.2 nm), nanoparticles on the surface	Increased hydrophobicity due to rough surface (Contact angle increased by 13⁰ compared to pure PVDF)	Higher antibacterial performance at lower concentrations (99.98% sterilization)
	PVDF-ZnO-Nanoclay	More uniform surface, nanoparticles embedded within matrix	Reduced surface roughness due to better dispersion (11.7 nm)	Moderate antibacterial efficiency, mainly due to ROS and Zn^2+^ ion release
Hydrophilicity	PVDF-Ag-Nanoclay	More hydrophobic	Increased contact angle and surface roughness (29.3 nm roughness at 1wt%)	Decreased water flux due to increased hydrophobicity (−7% at 1wt%)
	PVDF-ZnO-Nanoclay	Less hydrophilic	Hydrophilicity influenced by nanoparticle distribution	Lower water flux at 3wt% (−12%), enhanced antibacterial due to ROS

## Data Availability

Data are contained within the article and Supplementary Materials.

## Supplementary Materials

The following supporting information can be downloaded at: https://www.mdpi.com/article/10.3390/polym17081120/s1.

## References

[1] World Health Organization (2019). Progress on Household Drinking Water, Sanitation and Hygiene 2000–2017: Special Focus on Inequalities.

[2] Qu X., Brame J., Li Q., Alvarez P. (2012). Nanotechnology for a Safe and Sustainable Water Supply: Enabling Integrated Water Treatment and Reuse. Accounts Chem. Res..

[3] Khalaf A., Abu-Dalo D., AlShamaileh E. (2024). Synthesis, Characterization, and Application of Fe_2_O_3_ Nanophotocatalyst for the Treatment of Various Pollutants in Aqueous Phase: A Systematic Review. Sci. World J..

[4] Elkacmi R., Bennajah M. (2019). Advanced oxidation technologies for the treatment and detoxification of olive mill wastewater: A general review. J. Water Reuse Desalination.

[5] Dwairi I.M. (1991). Evaluation of Jordanian Phillipsite Tuff in Removal of Ammonia from Wastewater Experimental Study. Al-Balqa J..

[6] Rashid R., Shafiq I., Akhter P., Iqbal M., Hussain M. (2021). A state-of-the-art review on wastewater treatment techniques: The effectiveness of adsorption method. Environ. Sci. Pollut. Res..

[7] Gupta V.K., Ali I. (2013). Water Treatment by Membrane Filtration Techniques. Environmental Water.

[8] Cheng Y., Xia C., Garalleh H.A., Garaleh M., Chi N.T.L., Brindhadevi K. (2023). A review on optimistic development of polymeric nanocomposite membrane on environmental remediation. Chemosphere.

[9] Ji J., Liu F., Hashim N.A., Abed M.R.M., Li K. (2015). Poly(vinylidene fluoride) (PVDF) membranes for fluid separation. React. Funct. Polym..

[10] Lai C.Y., Groth A., Gray S., Duke M. (2014). Nanocomposites for Improved Physical Durability of Porous PVDF Membranes. Membranes.

[11] Nor N.A.M., Jaafar J., Ismail A.F., Mohamed M.A., Rahman M.A., Othman M.H.D., Lau W.J., Yusof N. (2016). Preparation and performance of PVDF-based nanocomposite membrane consisting of TiO_2_ nanofibers for organic pollutant decomposition in wastewater under UV irradiation. Desalination.

[12] Hong J., He Y. (2012). Effects of nano sized zinc oxide on the performance of PVDF microfiltration membranes. Desalination.

[13] Habba Y.G., Capochichi-Gnambodoe M., Serairi L., Leprince-Wang Y. (2016). Enhanced photocatalytic activity of ZnO nanostructure for water purification. Phys. Status Solidi (b).

[14] Taha A.K.T., Amir, Korkmaz A.D., Al-Messiere M.A., Baykal A., Karakuş S., Kilislioglu A. (2018). Development of Novel Nano-ZnO Enhanced Polymeric Membranes for Water Purification. J. Inorg. Organomet. Polym. Mater..

[15] Abdulhameed A., Al Omari R., Younes M., Algburi S. (2024). Cross-linked chitosan-adipic/zirconia (ZrO_2_) nanocomposite for removal of eosin y dye: Insight into physicochemical properties, adsorption modelling, isotherms, and kinetics. Int. J. Environ. Anal. Chem..

[16] Al-Maliki R.M., Alsalhy Q.F., Al-Jubouri S., Salih I.K., AbdulRazak A.A., Shehab M.A., Németh Z., Hernadi K. (2022). Classification of Nanomaterials and the Effect of Graphene Oxide (GO) and Recently Developed Nanoparticles on the Ultrafiltration Membrane and Their Applications: A Review. Membranes.

[17] Ma C., Hu J., Sun W., Ma Z., Yang W., Wang L., Ran Z., Zhao B., Zhang Z., Zhang H. (2020). Graphene oxide-polyethylene glycol incorporated PVDF nanocomposite ultrafiltration membrane with enhanced hydrophilicity, permeability, and antifouling performance. Chemosphere.

[18] Cheng W., Lu X., Kaneda M., Zhang W., Bernstein R., Ma J., Elimelech M. (2019). Graphene Oxide Functionalized Membranes: The Importance of Nanosheet Surface Exposure for Biofouling Resistance. Environ. Sci. Technol..

[19] Alnairat N., Dalo M.A., Abu-Zurayk R., Mallouh S.A., Odeh F., Al Bawab A. (2021). Green Synthesis of Silver Nanoparticles as an Effective Antibiofouling Material for Polyvinylidene Fluoride (PVDF) Ultrafiltration Membrane. Polymers.

[20] Mecha A.C., Chollom M.N., Babatunde B.F., Tetteh E.K., Rathilal S. (2023). Versatile Silver-Nanoparticle-Impregnated Membranes for Water Treatment: A Review. Membranes.

[21] Lai C.Y., Groth A., Gray S., Duke M. (2014). Enhanced abrasion resistant PVDF/nanoclay hollow fibre composite membranes for water treatment. J. Membr. Sci..

[22] Ma W., Li Y., Gao S., Cui J., Qu Q., Wang Y., Huang C., Fu G.-D. (2020). Self-Healing and Superwettable Nanofibrous Membranes with Excellent Stability toward Multifunctional Applications in Water Purification. ACS Appl. Mater. Interfaces.

[23] Abu-Zurayk R., Alnairat N., Bozeya A., Khalaf A., Abu-Dalo D. (2024). Enhanced properties of PVDF membranes using green Ag-nanoclay composite nanoarchitectonics. Mater. Res. Express.

[24] Al-Khaial M.Q., Chan S.Y., Abu-Zurayk R.A., Alnairat N. (2024). Biosynthesis and Characterization of Zinc Oxide Nanoparticles (ZnO-NPs) Utilizing Banana Peel Extract. Inorganics.

[25] Mahdy S.A., Raheed Q.J., Kalaichelvan P. (2012). Antimicrobial activity of zero-valent iron nanoparticles. Int. J. Mod. Eng. Res..

[26] (2011). Measurement of Antibacterial Activity on Plastics and Other Non-Porous Surfaces.

[27] Huang L., Zhao S., Wang Z., Wu J., Wang J., Wang S. (2016). In situ immobilization of silver nanoparticles for improving permeability, antifouling and anti-bacterial properties of ultrafiltration membrane. J. Membr. Sci..

[28] Szczyglewska P., Feliczak-Guzik A., Nowak I. (2023). Nanotechnology–General Aspects: A Chemical Reduction Approach to the Synthesis of Nanoparticles. Molecules.

[29] Alsoud A., Daradkeh S.I., Al-Bashaish S.R., Shaheen A.A., Jaber A.M.D., Abuamr A.M., Mousa M.S., Holcman V. (2024). Electrical Characterization of Epoxy Nanocomposite under High DC Voltage. Polymers.

[30] Zhao Q., Fu L., Jiang D., Ouyang J., Hu Y., Yang H., Xi Y. (2019). Nanoclay-modulated oxygen vacancies of metal oxide. Commun. Chem..

[31] Ye J., Li X., Hong J., Chen J., Fan Q. (2015). Photocatalytic degradation of phenol over ZnO nanosheets immobilized on montmorillonite. Mater. Sci. Semicond. Process..

[32] Awwad A., Abdeen A. (2013). Green synthesis of silver nanoparticles using carob leaf extract and its antibacterial activity. Int. J. Ind. Chem..

[33] Vanaja M., Gurusamy A. (2012). Coleus aromaticus leaf extract mediated synthesis of silver nanoparticles and its bactericidal activity. Appl. Nanosci..

[34] Assayed G.A.I., Shaheen A.A., Alsoud A., Al-Bashaish S.R., Mousa M.S., Knápek A., Sobola D. (2024). Structural and electrical characterization of cadmium phosphate glasses doped with different concentration of sodium chloride. Phys. Scr..

[35] Naqvi S.M.A., Soleimani H., Yahya N., Irshad K. (2014). Structural and optical properties of chromium doped zinc oxide nanoparticles synthesized by sol-gel method. AIP Conf. Proc..

[36] Ashraf R., Riaz S., Kayani Z.N., Naseem S. (2015). Effect of Calcination on Properties of ZnO Nanoparticles. Mater. Today Proc..

[37] Ahmad T., Pandey V., Husain M.S., Adiba, Munjal S. (2022). Structural and spectroscopic analysis of pure phase hexagonal wurtzite ZnO nanoparticles synthesized by sol-gel. Mater. Today Proc..

[38] Inoue M., Hirasawa I. (2013). The relationship between crystal morphology and XRD peak intensity on CaSO_4_ 2H_2_O. J. Cryst. Growth.

[39] Zhang B., Li J., Hu J., Liu L. (2021). Theory of polymer diffusion in polymer-nanoparticle mixtures: Effect of nanoparticle concentration and polymer length. Soft Matter.

[40] Prihandana G., Sriani T., Muthi’ah A., Machmudah A., Mahardika M., Miki N. (2022). Study Effect of nAg Particle Size on the Properties and Antibacterial Characteristics of Polysulfone Membranes. Nanomaterials.

[41] Roshani R., Ardeshiri F., Peyravi M., Jahanshahi M. (2018). Highly permeable PVDF membrane with PS/ZnO nanocomposite incorporated for distillation process. RSC Adv..

[42] Emadzadeh D., Lau W.J., Matsuura T., Rahbari-Sisakht M., Ismail A.F. (2014). A novel thin film composite forward osmosis membrane prepared from PSf–TiO_2_ nanocomposite substrate for water desalination. Chem. Eng. J..

[43] Razmjou A., Mansouri J., Chen V. (2011). The effects of mechanical and chemical modification of TiO_2_ nanoparticles on the surface chemistry, structure and fouling performance of PES ultrafiltration membranes. J. Membr. Sci..

[44] Shen J., Ruan H., Wu L., Gao C. (2011). Preparation and characterization of PES–SiO_2_ organic–inorganic composite ultrafiltration membrane for raw water pretreatment. Chem. Eng. J..

[45] Vatanpour V., Madaeni S.S., Khataee A.R., Salehi E., Zinadini S., Monfared H.A. (2012). TiO_2_ embedded mixed matrix PES nanocomposite membranes: Influence of different sizes and types of nanoparticles on antifouling and performance. Desalination.

[46] Rahimpour A., Madaeni S.S., Taheri A.H., Mansourpanah Y. (2008). Coupling TiO_2_ nanoparticles with UV irradiation for modification of polyethersulfone ultrafiltration membranes. J. Membr. Sci..

[47] Li J.-B., Zhu J.-W., Zheng M.-S. (2007). Morphologies and properties of poly(phthalazinone ether sulfone ketone) matrix ultrafiltration membranes with entrapped TiO_2_ nanoparticles. J. Appl. Polym. Sci..

[48] Li H., Wu H., Zhang W., Zhao X., Zhang L., Gao Y. (2021). Rheological mechanism of polymer nanocomposites filled with spherical nanoparticles: Insight from molecular dynamics simulation. Polymer.

[49] Ardeshiri F., Salehi S., Peyravi M., Jahanshahi M., Amiri A., Rad A. (2018). PVDF membrane assisted by modified hydrophobic ZnO nanoparticle for membrane distillation. Asia-Pacific J. Chem. Eng..

[50] Khulbe K.C., Feng C., Matsuura T., Kapantaidakis G.C., Wessling M., Koops G.H. (2003). Characterization of polyethersulfone-polyimide hollow fiber membranes by atomic force microscopy and contact angle goniometery. J. Membr. Sci..

[51] Mierzwa J.C., Arieta V., Verlage M., Carvalho J., Vecitis C.D. (2013). Effect of clay nanoparticles on the structure and performance of polyethersulfone ultrafiltration membranes. Desalination.

[52] Li J., Chen S., Liu W., Fu R., Tu S., Zhao Y., Dong L., Yan B., Gu Y. (2019). High Performance Piezoelectric Nanogenerators Based on Electrospun ZnO Nanorods/Poly(vinylidene fluoride) Composite Membranes. J. Phys. Chem. C.

[53] Hwang H.-Y., Kim D.-J., Kim H.-J., Hong Y.-T., Nam S.-Y. (2011). Effect of nanoclay on properties of porous PVdF membranes. Trans. Nonferrous Met. Soc. China.

[54] He Y., Hong J.M. (2011). Effect of Nano-Sized ZnO Particle Addition on PVDF Ultrafiltration Membrane Performance. Adv. Mater. Res..

[55] Pirtarighat S., Ghannadnia M., Baghshahi S. (2019). Green synthesis of silver nanoparticles using the plant extract of Salvia spinosa grown in vitro and their antibacterial activity assessment. J. Nanostruct. Chem..

[56] Abdelghany T.M., Al-Rajhi A.M.H., Al Abboud M.A., Alawlaqi M.M., Magdah A.G., Helmy E.A.M., Mabrouk A.S. (2018). Recent advances in green synthesis of silver nanoparticles and their applications: About future directions. A review. BioNanoScience.

[57] Parvataneni R. (2020). Biogenic synthesis and characterization of silver nanoparticles using aqueous leaf extract of Scoparia dulcis L. and assessment of their antimicrobial property. Drug Chem. Toxicol..

[58] Ganash M., Ghany T.A., Omar A. (2018). Morphological and biomolecules dynamics of phytopathogenic fungi under stress of silver nanoparticles. BioNanoScience.

[59] Girase B., Depan D., Shah J.S., Xu W., Misra R.D.K. (2011). Silver–clay nanohybrid structure for effective and diffusion-controlled antimicrobial activity. Mater. Sci. Eng. C.

[60] Roy A., Butola B., Joshi M. (2017). Synthesis, characterization and antibacterial properties of novel nano-silver loaded acid activated montmorillonite. Appl. Clay Sci..

[61] Sawai J., Shoji S., Igarashi H., Hashimoto A., Kokugan T., Shimizu M., Kojima H. (1998). Hydrogen peroxide as an antibacterial factor in zinc oxide powder slurry. J. Ferment. Bioeng..

[62] Atmaca S., Gül K., Cicek R. (1998). The effect of zinc on microbial growth. Turk. J. Med. Sci..

[63] Li Y., Liao C., Tjong S.C. (2020). Recent Advances in Zinc Oxide Nanostructures with Antimicrobial Activities. Int. J. Mol. Sci..

[64] Morrison K.D., Underwood J.C., Metge D.W., Eberl D.D., Williams L.B. (2014). Mineralogical variables that control the antibacterial effectiveness of a natural clay deposit. Environ. Geochem. Health.

[65] Agnihotri S., Bajaj G., Mukherji S., Mukherji S. (2015). Arginine-assisted immobilization of silver nanoparticles on ZnO nanorods: An enhanced and reusable antibacterial substrate without human cell cytotoxicity. Nanoscale.

[66] Jaber A.M., Zahra J.A., El-Abadelah M.M., Al-Mahadeen M.M., Sabri S.S., Kasabri V., Haddadin R.N. (2024). Evaluation of Spirooxindole-3,3’-pyrrolines-incorporating Isoquinoline Motif as Antitumor, Anti-Inflammatory, Antibacterial, Antifungal, and Antioxidant Agents. Anti-Inflamm. Anti-Allergy Agents Med. Chem..

[67] He W., Kim H.-K., Wamer W.G., Melka D., Callahan J.H., Yin J.-J. (2014). Photogenerated charge carriers and reactive oxygen species in ZnO/Au hybrid nanostructures with enhanced photocatalytic and antibacterial activity. J. Am. Chem. Soc..

[68] Hui A., Dong S., Kang Y., Zhou Y., Wang A. (2019). Hydrothermal Fabrication of Spindle-Shaped ZnO/Palygorskite Nanocomposites Using Nonionic Surfactant for Enhancement of Antibacterial Activity. Nanomaterials.

[69] Wang W., Li Y., Wang W., Gao B., Wang Z. (2019). Palygorskite/silver nanoparticles incorporated polyamide thin film nanocomposite membranes with enhanced water permeating, antifouling and antimicrobial performance. Chemosphere.

[70] Abdulhameed A., Al Omari R., Younes M., Algburi S. (2024). Chitosan polymer/functionalised rambutan peel using carboxylic acid: Process optimisation using Box-Behnken Design for brilliant green dye removal. Int. J. Environ. Anal. Chem..

[71] Gabriel J.S., Gonzaga V.A.M., Poli A.L., Schmitt C.C. (2017). Photochemical synthesis of silver nanoparticles on chitosans/montmorillonite nanocomposite films and antibacterial activity. Carbohydr. Polym..

[72] Al Hunaiti A., Ghazzy A., Aqel H., Abu Thiab T., Saeed R., Taha M., Hwaitat E., Zihlif M.Z., Imraish A. (2024). New Chitosan-Based Trimetallic Cu0.5Zn0.5Fe2O4 Nanoparticles: Preparation, Characterization, and Anti-cancer Activity Original Research Article distributed under the Creative Commons Attribution-NonCommercial-NoDerivs 4.0 International (CC BY-NC-ND 4.0) license. Pharm. Pract..

[73] Tian G., Huang Z., Wang H., Cui C., Zhang Y. (2022). Polycaprolactone nanofiber membrane modified with halloysite and ZnO for anti-bacterial and air filtration. Appl. Clay Sci..

[74] Ayyaru S., Dinh T.T.L., Ahn Y.-H. (2020). Enhanced antifouling performance of PVDF ultrafiltration membrane by blending zinc oxide with support of graphene oxide nanoparticle. Chemosphere.

[75] Li J.-H., Shao X.-S., Zhou Q., Li M.-Z., Zhang Q.-Q. (2013). The double effects of silver nanoparticles on the PVDF membrane: Surface hydrophilicity and antifouling performance. Appl. Surf. Sci..

[76] Abdul-Majeed M.A. (2018). Preparation and Characterization of AgNp/PVDF Cmposite Ultrafiltration Membrane. J. Eng..

[77] Kamaludin R., Majid L.A., Othman M.H.D., Mansur S., Kadir S.H.S.A., Wong K.Y., Khongnakorn W., Puteh M.H. (2022). Polyvinylidene Difluoride (PVDF) Hollow Fiber Membrane Incorporated with Antibacterial and Anti-Fouling by Zinc Oxide for Water and Wastewater Treatment. Membranes.

[78] Jia H., Wu Z., Liu N. (2017). Effect of nano-ZnO with different particle size on the performance of PVDF composite membrane. Plast. Rubber Compos..

