# Supplementary material for: Polyvinylidene Fluoride (PVDF) and Nanoclay Composites’ Mixed-Matrix Membranes: Exploring Structure, Properties, and Performance Relationships
Source: Polymers (Basel). 2025 Apr 20;17(8):1120. doi: 10.3390/polym17081120 (PMC12030574; doi:10.3390/polym17081120)
Supplement: Supplementary file 1 [file polymers-17-01120-s001.zip › polymers-3519840-supplementary.pdf]

Contact angles

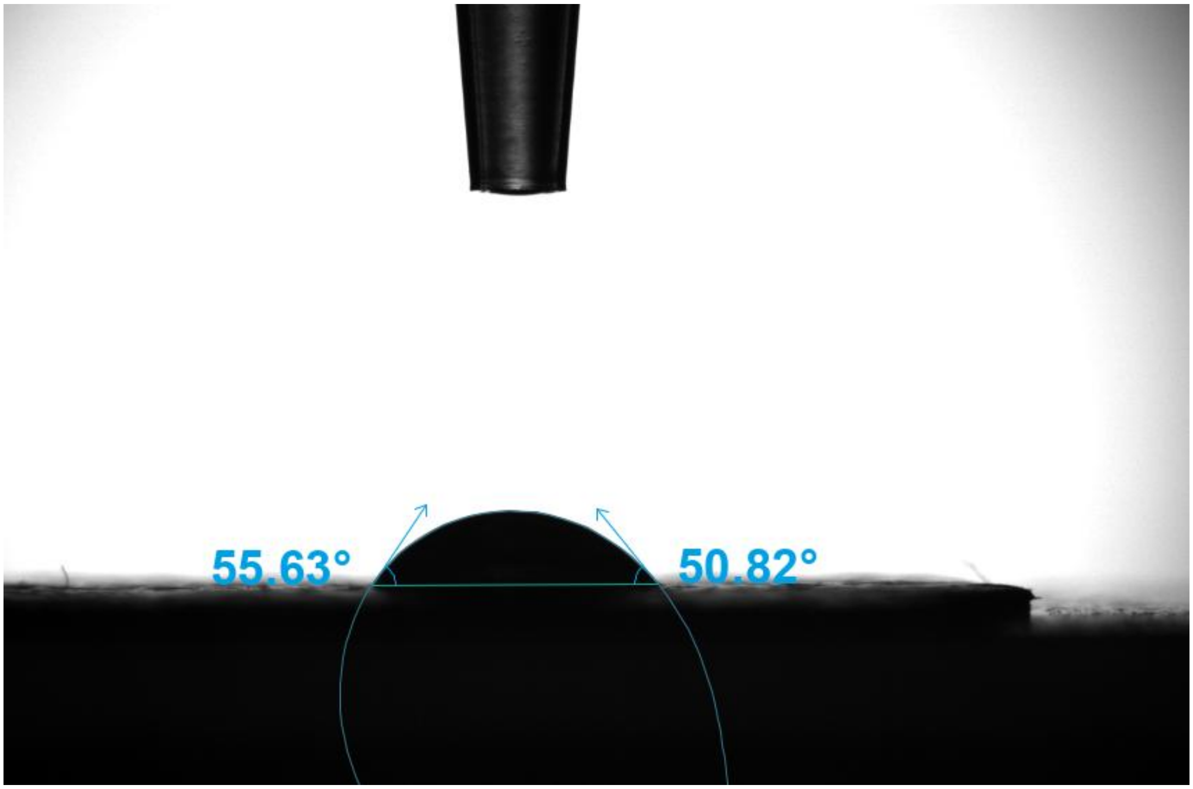

PVDF-pure

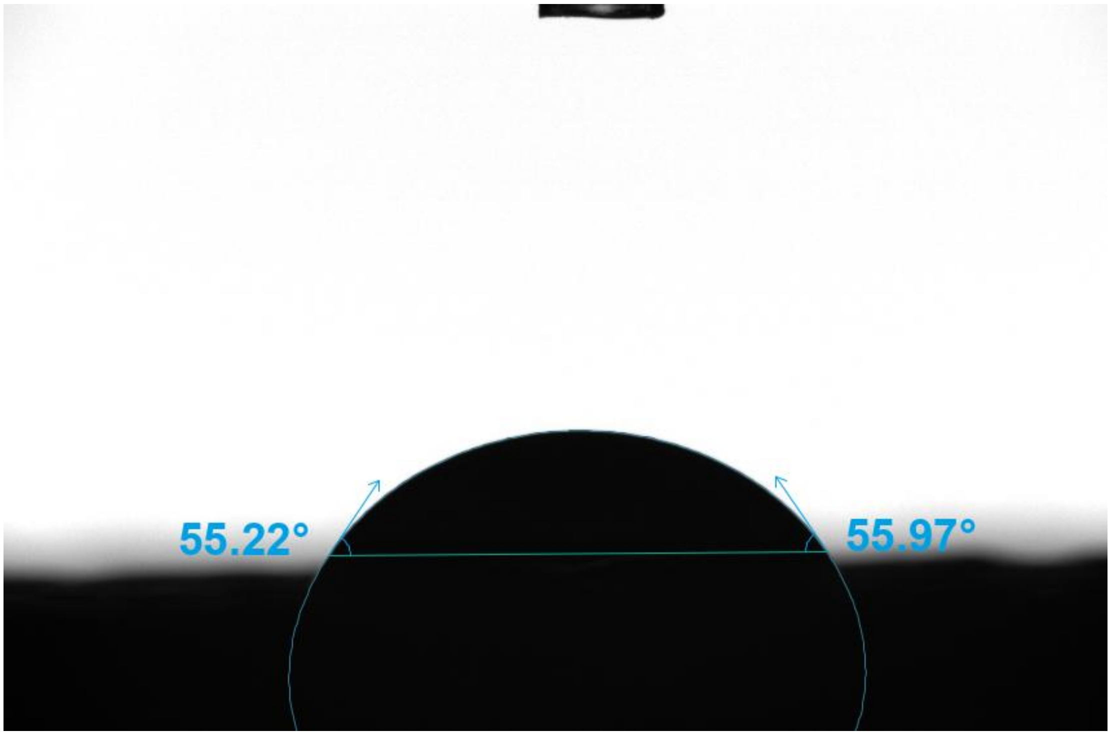

PVDF-1wt%Ag-Nanoclay

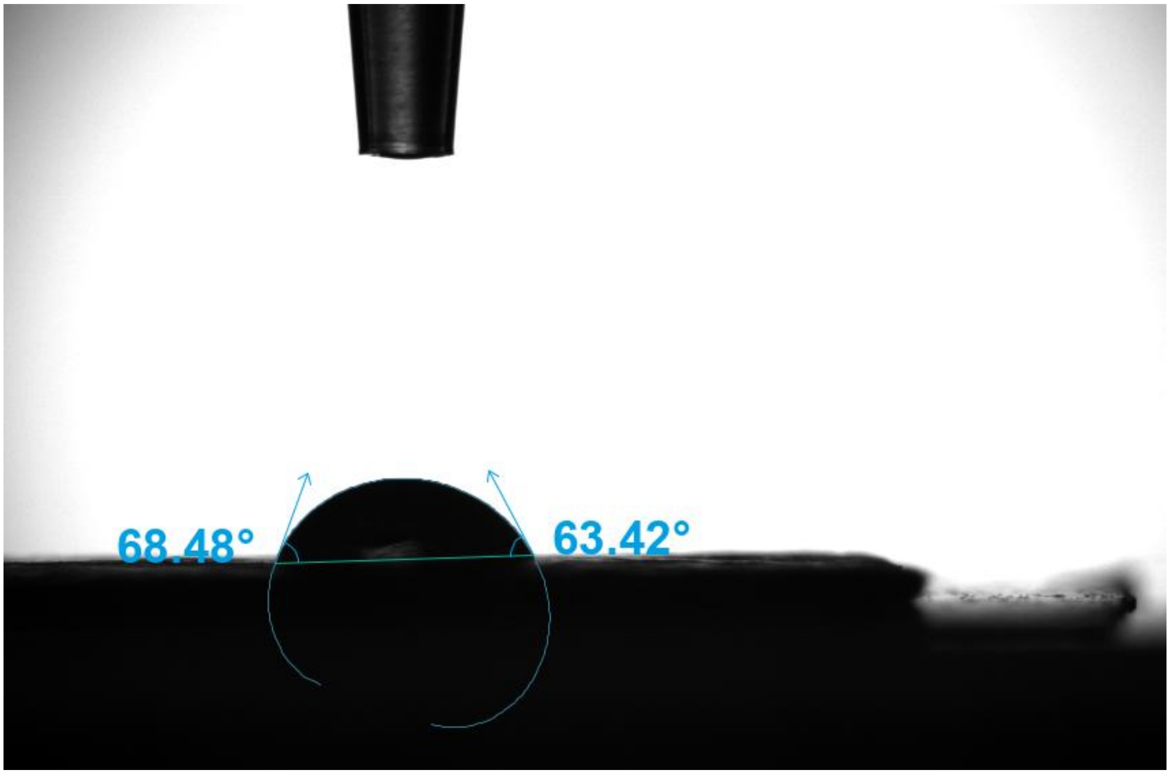

PVDF-3wt%Ag-Nanoclay

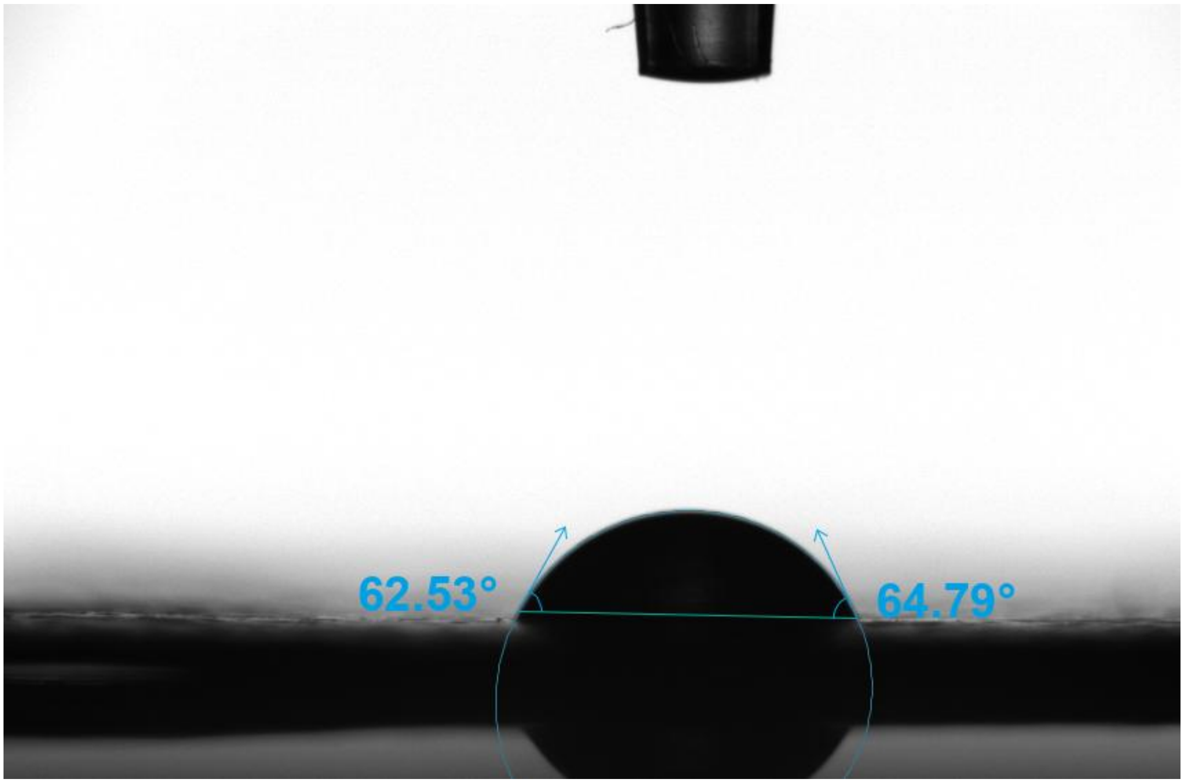

PVDF-1wt% ZnO-Nanoclay

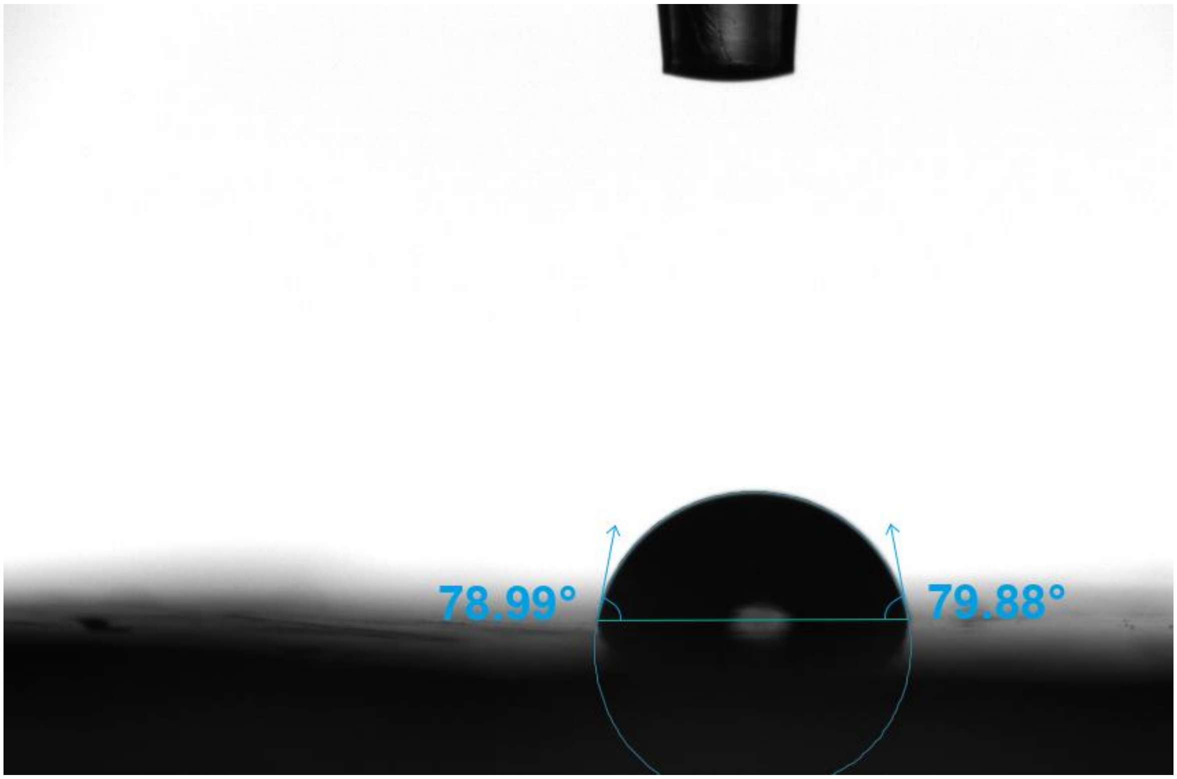

PVDF-3wt% ZnO-Nanoclay
